# Are the differences between intra-line and return-sweep fixation durations driven by linguistic, oculomotor, or visual processing? A comparison of eye movements during reading and z-string scanning

**DOI:** 10.3758/s13423-025-02738-x

**Published:** 2025-07-25

**Authors:** Adam J. Parker, Muchan Tao, Martin R. Vasilev

**Affiliations:** 1 Department of Experimental Psychology, Division of Psychology and Language Sciences, University College London, 26 Bedford Way, WC1H 0AP, London, UK; 2 Department of Language and Cognition, Division of Psychology and Language Sciences, University College London, Chandler House, 2 Wakefield Street, WC1N 1PF, London, UK

**Keywords:** Eye movements, Reading, Z-string scanning, Return-sweeps, Oculomotor coordination

## Abstract

Return-sweeps, which move the reader’s gaze from the end of one line to the beginning of the next, typically result in shorter line-final fixations and longer accurate line-initial fixations compared to intra-line fixations. The mechanisms underlying these differences have been the subject of debate. To assess the linguistic and oculomotor contributions to these return-sweep fixation differences, we compared the eye movements of 41 participants during reading and z-string scanning, an oculomotor control condition that is devoid of useful linguistic content. Our results indicate that line-final fixations are shorter than intra-line fixations, while accurate line-initial fixations are longer than intra-line fixations, under both tasks, underscoring the significant role of the oculomotor system in determining fixation durations across tasks. Notably, the reduction in line-final fixation durations compared to intra-line fixations did not differ between tasks. This suggests that oculomotor coordination or visual processing, rather than linguistic processing, drives shorter line-final fixations. In contrast, the difference in the increase in duration for accurate line-initial fixations between reading and z-string scanning implies that longer accurate line-initial fixations are likely a result of lexical processing, oculomotor coordination, and visual processing. These findings advance our understanding of eye movement control by highlighting the combined influence of linguistic and oculomotor processes on return-sweep fixation durations.

**Supplementary Information:**

The online version contains supplementary material available at 10.3758/s13423-025-02738-x.

In recent years, research on return-sweep saccades during reading has significantly increased (Adedeji, Vasilev, Kirkby, & Slattery 2022; Christofalos et al., 2024; Parker, Kirkby, & Slattery, 2017; Parker, Slattery, & Kirkby, 2019a; Parker, Nikolova, Slattery, Liversedge, & Kirkby, 2019b; Parker & Slattery, 2019, 2021, 2024; Parker, Kirkby, & Slattery, 2020; Parker, Ryäsyänen, & Slattery, 2023; Slattery & Parker, 2019; Slattery & Vasilev, 2019; Vasilev, Adedeji, Laursen, Budka, & Slattery, 2021; Wang et al., 2024). Return-sweeps are large saccadic eye movements that move readers’ gaze from one line to the next, significantly impacting fixation durations before and after the return-sweep. Consistent findings show that line-final fixations (fixations just before a return-sweep) are shorter than intra-line fixations (fixations within a line), while accurate line-initial fixations (fixations at the start of a line followed by a rightwards saccade) are longer than intra-line fixations (Abrams & Zuber, 1972; Hawley, Stern, & Chen, 1974; Heller, 1982; Hofmeister, 1998; Rayner, 1977, 1978). Several accounts have been proposed to explain these differences in fixation duration, with explanations being described as lexical processing accounts or oculomotor/visual accounts. To date, no study has examined how lexical processing and oculomotor coordination/visual processing contribute to shorter line-final fixations and longer accurate line-initial fixations (relative to intra-line fixations). To this end, we compared participants’ eye movements as they read multi-line texts and scanned rows of letter strings under a *z-reading paradigm* (Vitu, O’Regan, Inhoff, & Topolski, 1995), an oculomotor control condition devoid of useful linguistic content. If differences in fixation duration across fixation types were the same across both tasks, then this would indicate that return-sweep fixation duration differences are the consequence of oculomotor coordination or visual processing rather than lexical processing. This is because z-strings do not contain useful lexical information, so fixation durations would be determined by oculomotor control and letter perception, which is shared across both tasks.

Return-sweep saccades typically launch from 4–8 characters from the line’s end in alphabetic reading (Hofmeister et al., 1999; Parker & Slattery, 2019; Parker et al., 2020; Rayner, 1998). Numerous studies have confirmed that line-final fixations are shorter than intra-line fixations (Abrams & Zuber, 1972; Adedeji et al., 2022; Christofalos et al., 2024; Hawley et al., 1974; Heller, 1982; Parker et al., 2019a, b; Parker & Slattery, 2021; Parker et al., 2023; Rayner, 1977; Slattery & Parker, 2019; Vasilev et al., 2021; Wang et al., 2024). It has been suggested that these shorter fixations result from return-sweep preparation, with the primary purpose of line-final fixations being to orient the visual system. An extreme version of this *linguistic account* posits that line-final fixations do not involve linguistic processing. Supporting this, Hofmeister (1998) found that text degradation did not affect line-final fixation durations. However, ample evidence now suggests otherwise, as lexical frequency has been shown to influence line-final fixations and reading times on line-final words (Parker et al., 2023; Parker & Slattery, 2024).

Alternative explanations for shorter line-final fixations have been proposed. Rayner (1977) suggested that the absence of a word to the right of fixation eliminates the need to process parafoveal information, thus shortening line-final fixations. This, in part, could reflect a skipping cost, where skipping costs refer to the additional time required when a reader processes a word before skipping over the next one. According to the E-Z Reader model (Reichle & Drieghe, 2013), fixations before a skipped word tend to be longer because the reader engages in parafoveal processing of the upcoming word before deciding whether to skip it. At the end of a line, this process is disrupted. Since there is no upcoming word to the right of the line-final word, readers do not engage in parafoveal processing in the same way as they would during intra-line reading. As a result, skipping costs are effectively eliminated, leading to shorter fixation durations on line-final words. Similarly, shorter line-final fixations could reflect reduced lateral masking at the end of a line (Parker & Slattery, 2024). While it is difficult to classify parafoveal processing/skipping cost accounts as being an oculomotor, visual, or linguistic account, given the complex interactions between these systems during parafoveal processing, the latter lateral masking account can be classified as an *oculomotor/visual account*. Alternatively, a *linguistic account* suggests that readers may terminate line-final fixations earlier as they can conduct additional lexical processing during the return-sweep, which is longer than typical intra-line reading saccades, and this results in shorter line-final fixations (see Parker & Slattery, 2024, for a discussion).

Return-sweeps, like any saccade, are prone to systematic and random error (McConkie et al., 1988). They undershoot their target 40–60% of the time, necessitating an immediate corrective saccade towards the left margin (Slattery & Vasilev, 2019). Consequently, return-sweeps have two possible outcomes: accurate line-initial fixations, where the return-sweep is followed by a rightward saccade, or under-sweep fixations, where readers land short of their intended target and make a leftward corrective saccade before a rightward saccade.

Accurate line-initial fixations, which typically land 4–8 characters from the start of the line, are longer than intra-line fixations (Adedeji et al., 2022; Christofalos et al., 2024; Parker et al., 2019a, b; Parker & Slattery, 2021; Parker et al., 2020, 2023; Slattery & Parker, 2019; Wang et al., 2024). Several accounts have been proposed to explain this. In an account that involves lexical, oculomotor, and visual processing, Parker, Kirkby, and Slattery (2017) suggested that the absence of parafoveal preview for line-initial words, which lie outside the perceptual span prior to fixation, might result in longer line-initial fixations. Alternatively, Rayner (1978) and Kuperman, Dambacher, Nuthmann, and Kliegl (2010) put forward an *oculomotor/visual account* that suggests that longer accurate line-initial fixations might result from establishing a mode of saccadic programming after the return-sweep.

Under-sweep fixations are typically shorter than intra-line fixations and are generally assumed to involve little lexical processing (Hawley et al., 1974; Hofmeister, 1998; Shebilske, 1976). These fixations are thought to be primarily due to oculomotor error, with the main goal being to rapidly plan and execute a corrective saccade to the intended target of the return-sweep (Becker, 1976). While studies have reported that under-sweep fixation durations are not influenced by the properties of the fixated word (i.e., word length, lexical frequency, or cloze predictability; Parker et al., 2020; Slattery & Parker, 2019), there is evidence that readers utilize this pause before a corrective saccade to extract information from both the undershot line-initial word and the fixated word that facilitates subsequent processing (Parker & Slattery, 2019; Parker et al., 2020; Slattery & Parker, 2019). This is because fixations on a line-initial word are shorter if preceded by a fixation on the second word on a line. Previously, in a study comparing reading and letter scanning, Hofmeister (1998) reported that under-sweep durations did not differ between tasks, suggesting a minimal impact of lexical processing on under-sweep fixation durations.

The *z-reading paradigm* offers a way to assess linguistic and oculomotor/visual contributions to return-sweep fixation differences. In this paradigm, participants read strings of meaningless letters resembling real text (e.g., *“Eye movements during reading”*becomes *“Xxx xxxxxxxxx xxxxxx xxxxxxx”*), preserving the text’s spatial layout but removing higher-level linguistic information. This provides an excellent oculomotor control condition for reading. The *z-reading paradigm* has been associated with longer fixation durations (Al-Zanoon, Dambacher, & Kuperman, 2017; Gagl et al., 2022; Rayner & Fischer, 1996; Vitu et al., 1995), increased skipping for longer letter strings (Rayner & Fischer, 1996; Vitu et al., 1995), and fewer regressions (Nuthmann, Engbert, & Kliegl, 2007). The paradigm has previously been used to examine whether shorter under-sweep fixations are the result of general oculomotor coordination processes.

Hofmeister (1998) compared eye movements during reading and z-string scanning. Their analyses were primarily concerned with landing positions, where it was reported that initial landing positions of line-initial fixations were further from the margin during scanning than reading. They also compared under-sweep fixation durations, noting no differences between tasks, enabling Hofmeister to conclude that under-sweep fixations are almost exclusively governed by oculomotor control. Note, however, that Hofmeister did not compare line-final or accurate line-initial fixations between tasks. We, therefore, aimed to use the *z-reading paradigm* to differentiate between linguistic and oculomotor/visual contributions to return-sweep fixation duration differences.

## Research questions and predictions

We pre-registered the following predictions:

### Return-sweep fixation types during paragraph reading

Within our statistical modeling framework, we applied a coding scheme that enabled us first to compare return-sweep fixations with intra-line reading fixations during reading. Our questions and predictions for return-sweep fixation durations during reading are as follows:

- *Are line-final reading fixations shorter than intra-line reading fixations?* We predicted shorter line-final reading fixations relative to intra-line reading fixations.
- *Are accurate line-initial reading fixations shorter than intra-line reading fixations?* We predicted longer accurate line-initial reading fixations relative to intra-line reading fixations.
- *Are under-sweep reading fixations shorter than intra-line reading fixations?* We predicted shorter under-sweep reading fixations relative to intra-line reading fixations.

### Differences between fixation types between z-string scanning and multiline text reading

Within our statistical models, task type is coded to compare fixations during z-string scanning to multiline text reading. As such, our predictions are qualified by interactions within statistical models. Our questions and predictions are as follows:

- *Do intra-line reading fixation durations differ from intra-line z-string scanning fixation durations?* Previous studies have reported longer fixations during z-string scanning than during reading (e.g., Rayner & Fischer, 1996). Therefore, we predicted longer intra-line fixations during z-string scanning (i.e., a significant simple effect of task).
- *Does the reduction in duration for line-final fixations (relative to intra-line fixations) differ between reading and z-string scanning?* If shorter line-final fixations during reading result from lexical processing, then we anticipated similar durations between intra-line fixations and line-final fixations during scanning, which results in an interaction between fixation type and task. If, however, shorter line-final fixations during reading are driven by oculomotor coordination/visual processing, then we would expect shorter line-final fixations across both tasks and no interaction when comparing data across tasks.
- *Does the increase in duration for accurate line-initial fixations (relative to intra-line fixations) differ between reading and z-string scanning?* If longer accurate line-initial fixations during reading result from linguistic processing, we would expect similar durations between intra-line and accurate line-initial fixations during scanning, resulting in an interaction between fixation type and task. This is because readers will be able to engage in lexical processing at the start of a new line during reading, but not scanning. If, however, longer accurate line-initial fixations during reading are driven by oculomotor coordination/visual processing, then we would expect longer accurate line-initial fixations across both tasks and no interaction when comparing data across tasks.
- *Does the reduction in duration for under-sweep fixations (relative to intra-line fixations) differ between reading and z-string scanning?* If readers engage in lexical processing during an under-sweep fixation that facilitates their subsequent reading behavior (Parker & Slattery, 2019; Parker et al., 2020; Slattery & Parker, 2019), then we might observe a slight difference in the reduction in durations for under-sweep fixations relative to intra-line fixations across tasks (i.e., a significant interaction). However, given that under-sweep fixations are generally considered to be under oculomotor control (Hofmeister, 1998), we may alternatively observe similar reductions for under-sweep fixations (relative to intra-line fixations) across both tasks and no interaction when comparing data across tasks.

Additionally, we conducted exploratory analyses of return-sweep and corrective saccade parameters. The justification for these analyses was twofold: (1) they enabled us to compare the effect of task demands on return-sweep behavior, and (2) they facilitated interpretation of the data.

## Methods

This experiment was pre-registered on the Open Science Framework (OSF) before data collection. The task materials, analysis scripts, and anonymized data (https://osf.io/tpf8e/?view_only=96406d577b8241aeb98c1b0785349580) and the pre-registration template (https://osf.io/hxzjv/?view_only=c07d06f9a962421fa9dbd79fe33ab600) are available on the Open Science Framework.

### Participants

*A priori* power analyses were conducted for all fixed effects of interest for our comparison of reading and scanning fixations within a frequentist linear mixed-effects modelling framework. We started by simulating multi-level data for 40 statistical subjects, where each statistical subject had data for 30 trials of text reading and 30 trials of z-string scanning, and characters were displayed across four lines in each trial. The fixation durations for each fixation type during text reading were taken from Parker and Slattery’s (2021) short line condition as the line lengths were comparable: intra-line fixations: 200.6 ms, line-final fixations: 191.4 ms, accurate line-initial fixations: 257.9 ms, and under-sweep fixations: 148.9 ms. For z-string scanning, we simulated a 38-ms increase in fixation duration for intra-line reading (Rayner & Fischer, 1996) such that the simulated duration equated to approximately 238.6 ms. Under the oculomotor account, we would expect that return-sweep fixations should not differ from intra-line fixations during scanning. Hence, we simulated data showing a negligible effect of fixation type on z-string scanning. We simulated this data 1000 times and, on each run, fitted a linear mixed-effects model to the data $(log 10 (fixation~duration) \sim \, fixation~type \times stimuli~type~+~(1~ {/}~participant)~+~(1~{/}~item))$, tallying each time a significant result was obtained for each fixed effect. Simulations suggested that 40 participants would provide sufficient power to detect all critical interactions where we predicted a difference with sufficient power (i.e., $>90\%$) using a significance threshold of $| {t}| > 2$.

To reach our pre-registered sample size, we initially recruited 55 participants via the UCL Psychology and Language Sciences SONA Participant Pool. Participants were aged between 18 and 45 years old, had spoken English for a minimum of 10 years, had no language, hearing, or visual impairments, and had no history of neurological illness. Participants were reimbursed at a rate of £9.00/hour or received course credit for their participation. We imposed several data cleaning procedures that resulted in a final sample of 41 participants. For more information on the data cleaning procedures, see “Data cleaning and final sample” section.

The experimental procedure was granted ethical approval by the UCL Department of Experimental Psychology’s Ethics Chair, ethics application number: EP_2021_015.

### Reading task

**Fig. 1:**
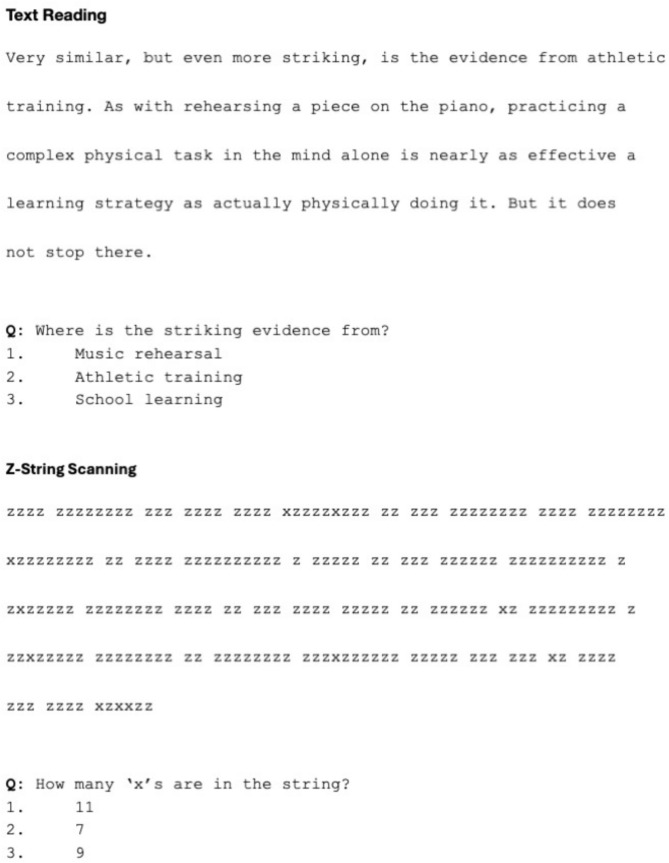
Example stimuli and question for text reading and z-string scanning

Thirty passages were taken from the Provo Corpus (Luke & Christianson, 2018). On average, the paragraphs were 49.97 words long ($$SD_{words} = 5.80; range_{words}:$$ 40–59). The mean word length was 4.75 letters ($$SD_{letters}= 2.51; range_{letters}:$$ 1-15). Words in each passage had an average Zipf frequency of 5.71 ($$SD_{zipf}= 1.42; range_{zipf}= $$ 1.17–7.67) based on the SUBTLEX-UK Corpus (Van Heuven, Mandera, Keuleers, & Brysbaert, 2014) and an average cloze probability of 0.20 ($$SD_{cloze}= 0.20; range_{cloze}$$= 0.00–1.00). Each paragraph was 2.63 sentences long on average ($$SD_{sentences}= 0.96; range_{sentences}:$$ 1–5 sentences) and was displayed across 4.13 lines on average ($$SD_{lines} 0.51; range_{lines}:$$ 3–5). During the passage reading task, participants were instructed to read silently for comprehension while their eye movements were recorded. After reading each paragraph, participants were asked a single comprehension question with three options (see Fig. 1).

### Scanning task

This scanning task is based on the *z-reading paradigm* (Vitu et al., 1995). The characters in the paragraphs from the Reading Task were replaced with the letter *z*, preserving capitalization and empty spaces (but not punctuation, to avoid distractors in the search task), such that words and z-strings were matched on length. The letter *x* was randomly inserted 5–15 times in the string of *z*s. Statistical analyses reported in the Online Supplemental Materials confirms that the placement of *x*s was approximately equally distributed across the spatial extent of the line. Participants were instructed to scan the string of letters (from left to right) and count how many times the letter *x* appears in it. After each trial, participants had to select the correct number of *x*s from three options.

### Apparatus

Eye movements were recorded using an SR Research EyeLink 1000-Plus eye-tracker, sampled at 1000 Hz. While viewing was binocular, only the right eye was tracked. To minimize head movements, a chin-and-forehead rest was used. Stimuli were presented on a 23.8” Dell G2422HS LCD monitor (resolution: $1920 \times 1080$) in 18 pt. Courier New font as black text over a white background. The eye-to-screen distance was 84 cm, such that each letter subtended $0.26^{\circ }$ horizontally. The experiment was programmed in SR Research Experiment Builder and was run on a Windows 11 PC.

### Procedure

The experiment started with a nine-point calibration and validation procedure. Calibration accuracy was kept at ${<}\,0.4^{\circ }$ across the experiment. Drift checks were presented before every trial, and participants were recalibrated whenever necessary, but at least every 15 trials. Participants were randomly allocated to complete the reading task first, followed by the scanning task or vice versa. For the scanning task, participants were explicitly instructed to adopt the same left-to-right strategy that is typical for English reading. Each task started with two practice trials, followed by 30 experimental trials. Participants were offered breaks whenever recalibration occurred. Each trial started with a fixation point that appeared to the left of the first character on the first line. Once a stable fixation was detected, the experimenter started the trial. When a participant had finished reading or scanning, they pressed the *space bar* to terminate the trial and then answered the multiple-choice question by pressing either *1*, *2*, or *3* on the keyboard.

### Data analysis

#### Data cleaning and final sample

We pre-registered that participants needed to score 70% or more on the reading comprehension questions. This led to the removal of six participants. We additionally removed one participant’s data as they failed to complete the study and a further seven due to calibration issues, excessive blinking, poor quality data, or corrupted files. The final sample consisted of 41 participants (31 female) with a mean age of 22.39 years (${SD}_{\text {years}}= 3.38$).

The data of the remaining 41 participants were pre-processed using the popEye package (version 0.8.3.1; Schro-eder, 2019) within R (version 4.4.3; R Development Core Team, 2020). Fixations were automatically vertically aligned against the text using the *chain* method (Carr, Pescuma, Furlan, Ktori, M., & Crepaldi, 2022). Fixations less than 50 ms were combined with the next fixation if they were within one character of each other. We then defined return-sweep fixations based on the following criteria: *Line-final fixations* were defined as the fixation occurring immediately before a return-sweep saccade (i.e., the last fixation on the line); *under-sweep fixations* were defined as the fixation immediately following a return-sweep saccade, which is then followed by a leftward (corrective) saccade; and *accurate line-initial fixations* were defined as the fixation immediately following a return-sweep saccade, which is then followed by a rightward saccade (i.e., in the reading direction).

Finally, we applied several pre-registered data-cleaning procedures prior to analysis. We pre-registered that we would remove trials in which participants made five or more blinks, leading to the removal of 20.81% of trials. For the remaining trials, fixations preceded or followed by a blink were removed, as were fixations that were shorter than 50 ms or longer than 1200 ms, resulting in the removal of 4.96% of fixations. We then applied a Hoaglin and Iglewicz (1987) outlier removal procedure to reading time data to identify outliers individually for each participant across each statistical condition. This procedure defined outliers as data points that were 2.2 times the difference between the first quartile (*Q1*) and the third quartile (*Q3*), above or below the *Q1* and *Q3* values (e.g., lower boundary = *Q1* – 2.2 $\times$ (*Q3*-*Q1*); upper boundary = *Q3* + 2.2 $\times$ (*Q3*-*Q1*)). This led to the removal of 1.88% of fixations.

#### Registered confirmatory analysis of return-sweep fixation duration

For our pre-registered analyses, a series of linear mixed-effects models were fitted to *log10* transformed data using the *lmer()* function from the lme4 package (version 1.1.36; Bates et al., 2015). The model comparing fixation durations between tasks initially adopted the structure *dv* $\sim$ *Task* $\times$ *Fixation Type +* (*1 + Task* $\times$ *Fixation Type / participant) +* (*1 + Task* $\times$ *Fixation Type / item*), where participant and item are random factors. Treatment coding was utilized so that data for intra-line fixations from the reading task represented the intercept to which return-sweep fixations across the two tasks were compared. To specifically examine return-sweep fixation durations during scanning, we fitted an additional exploratory model to scanning data: *dv* $\sim$ *Fixation Type + (1 + Fixation Type / participant) + (1 + Fixation Type / item)*. For all models, we report regression coefficients (*b*), standard errors (*SE*), and *t*-values.

To estimate the best-fitting random structure for each model, the *buildmer()* function from the buildmer package (version 2.11; Voeten, 2023) was used. First, a maximal structure was fitted to the data before applying a backwards elimination process based on the significance of the change in log likelihood between models. The most basic and possible model retained all fixed effects and random intercepts for participants and items.

To evaluate the evidence for the critical null effects, we supplemented our analyses with Bayes Factor analysis. Bayes factors quantify how much evidence the data (and priors) provide in favor of two competing models and allow us to infer how much a given hypothesis is consistent with the data (for reviews, see Nicenboim, Schad, & Vasishth, 2021, and Wagenmakers, 2017). Bayes factors were computed by first fitting Bayesian linear-mixed effects models to fixation duration data using the *brm()* function from the brms package (version 2.22.0; Bürkner, 2017). The models included the same fixed effects as the *lmer()* models. Non-informative priors *normal(0,1)* were assumed for each fixed effect. Each model used 12,000 iterations with four chains, where the first 2000 iterations were discarded due to warm-up. Then the *hypothesis()* function was implemented to calculate the Bayes factors (*BF*_10_) for each fixed effect. The *hypothesis()* function computes Bayes factors using the Savage–Dickey density ratio method (Dickey, 1971), where Bayes factors for individual parameters within a model are taken as the posterior density of the model parameter of interest divided by the prior density at the critical point of inference (e.g., zero if assessing whether an estimate is not equal to zero).

The combination of frequentist and Bayesian analysis enabled us to take a two-stage approach to inference. We considered results to be statistically significant where $| {t}| > 2$. If $| {t}| < 2$ and $BF_{10}> 1/3$, we considered there to be insufficient evidence. If $| {t} | < 2$ and $BF_{10}< 1/3$, we concluded that there was evidence in favor of the null hypothesis.

#### Non-registered exploratory analysis of return-sweep and corrective saccade parameters

For completeness, we analyzed several return-sweep and corrective saccade parameters: *return-sweep launch position* (character position relative to the end of the line), *probability of making an under-sweep fixation*, *landing position of accurate line-initial fixations* (character position relative to the start of the line), and *landing position of under-sweep fixations* (character position relative to the start of the line). For the analyses of launch position and landing positions only, we removed data points where the fixation was either more than 30 characters from the end of a line or more than 30 characters from the start of the line to exclude ends of an elongated tail of the distribution. We did not exclude these fixations from the pre-registered analysis of fixation duration. For line-final fixations, accurate line-initial fixations, and under-sweep fixations, this led to the removal of 13.73%, 6.22%, and 8.25% of fixations, respectively. For each measure, we fitted (generalized) linear mixed-effects models using the *(g)lmer()* function from the lme4 package. The model comparing parameters between reading and scanning was specified as *dv* $\sim$ *Task + (1 + Task / participant) + (1 + Task / item)*. Treatment coding was utilized so that data from the reading task represented the intercept to which scanning data were compared. As with our registered analyses, we used the *buildmer()* function to determine the random effects structure and combined frequentist and Bayes factor analysis to adopt a two-stage approach to inference.

## Results

### Task accuracy

Participants’ task accuracy was lower during reading 84.57% (*SD*= 36.13%) than during scanning 95.7% (*SD*= 20.39%), *b*= 1.23, *SE*= 0.39, *z*= 3.19, *BF*_10_= 34.63.

### Eye movements

#### Registered confirmatory analysis of return-sweep fixation duration

**Fig. 2:**
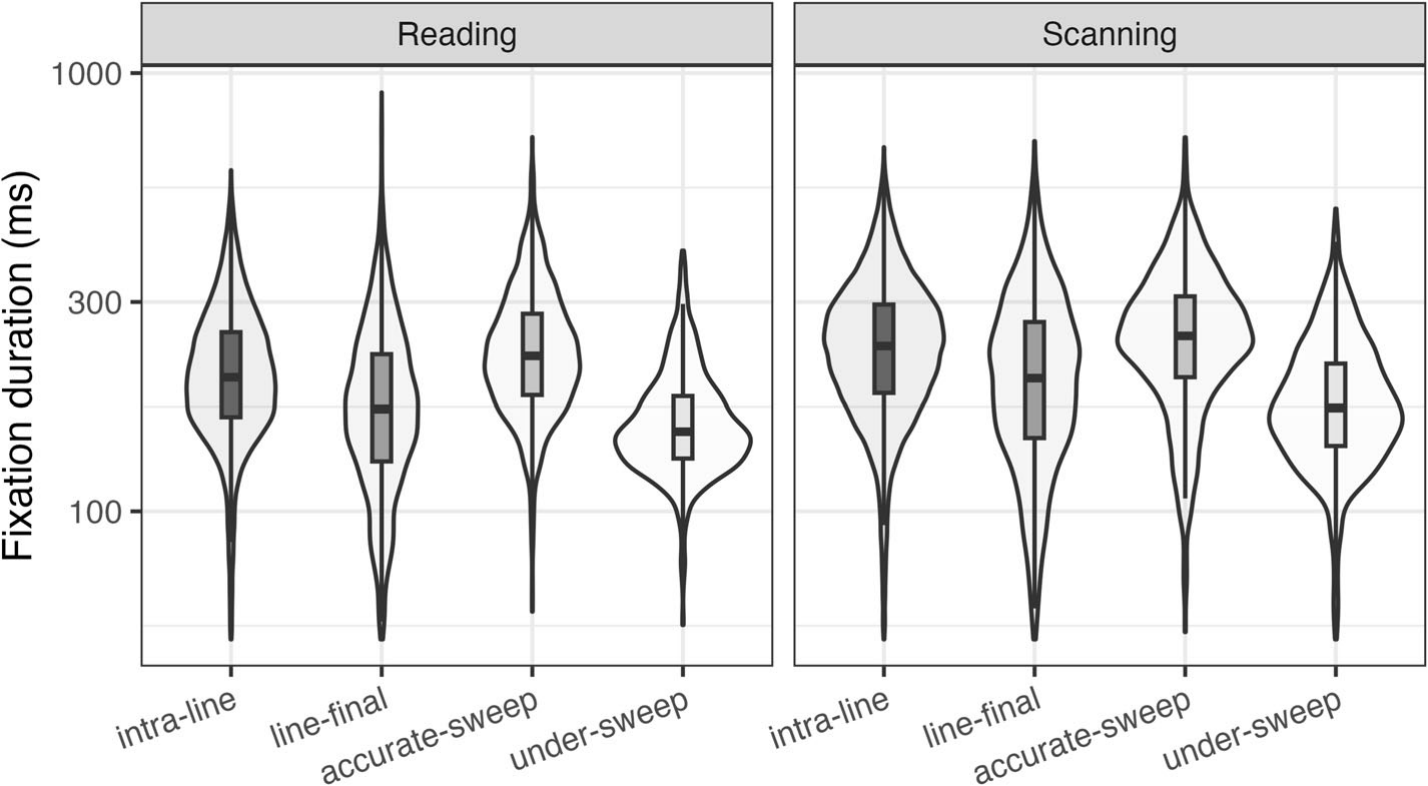
Return-sweep fixation durations per task. The box extends from the first to the third quartile, with the *line in the middle* representing the median

We report a confirmatory analysis of fixation durations where we compared return-sweep fixations to intra-line fixations across tasks. Mean fixation durations are reported in Table 1, and distributions are visualized in Fig. 2.

**Table 1:** Mean return-sweep fixation durations per task

Fixation type	Reading	Scanning
Intra-Line	216 (76)	248 (88)
Line-Final	186 (84)	216 (96)
Accurate Line-Initial	241 (81)	260 (92)
Under-Sweep	164 (48)	184 (63)

Note: ^a^Standard deviations are shown in parentheses

The model fitted to log-transformed fixation duration data (*lmer*(*dv*$\sim$ *Task* $\times$ *Fixation Type + (1 / Participant) + (1 + Task / Item)*)) indicated that line-final reading fixations and under-sweep reading fixations were shorter than intra-line reading fixations, while accurate line-initial reading fixations were longer than intra-line reading fixations (see Table 2). The simple effect of Task indicated that intra-line fixations were longer during scanning than reading. The difference between intra-line and line-final fixation durations did not differ between reading and scanning. However, interactions in the model indicate that the increase in duration of accurate line-initial fixations compared to intra-line fixations was smaller during scanning than reading and that the decrease in duration of under-sweep compared to intra-line fixations was greater for scanning than reading.

**Table 2:** Linear mixed-effects results and bayes factors for return-sweep fixation durations

Dataset	Fixed effect	b	SE	t	BF10
Reading and scanning	(Intercept)	2.30	0.01	309.62	−
	Task [Scanning]	0.06	<0.01	27.41	Inf
	Fixation type [Line-Final]	−0.08	<0.01	−33.32	Inf
	Fixation type [Accurate]	0.05	<0.01	17.98	Inf
	Fixation type [Under-Sweep]	−0.11	<0.01	−31.13	Inf
	Fixation type [Line-Final] $\times$ Task [Scanning]	0.01	<0.01	1.79	1.32e-02
	Fixation type [Accurate] $\times$ Task [Scanning]	−0.03	<0.01	−6.70	2.94e+17
	Fixation type [Under-Sweep] $\times$ Task [Scanning]	−0.01	<0.01	−2.50	1.16e-01
Scanning	(Intercept)	2.36	0.01	298.38	−
	Fixation type [Line-Final]	−0.07	<0.01	−29.05	2.27e+14
	Fixation type [Accurate]	0.03	<0.01	9.36	5.53e+18
	Fixation type [Under-Sweep]	−0.12	<0.01	−35.37	8.92e+13

To examine whether the difference between intra-line and accurate line-initial fixations was small but reliable or completely abolished for scanning, we fitted a supplemental model to scanning data *lmer*(*dv*$\sim$ *Fixation Type + (1 / Participant) + (1 / Item)*)). The model indicated that line-final and under-sweep fixations were shorter than intra-line fixations, and accurate line-initial fixations were longer than intra-line scanning fixations.

#### Non-registered exploratory analysis of return-sweep and corrective saccade parameters

**Fig. 3:**
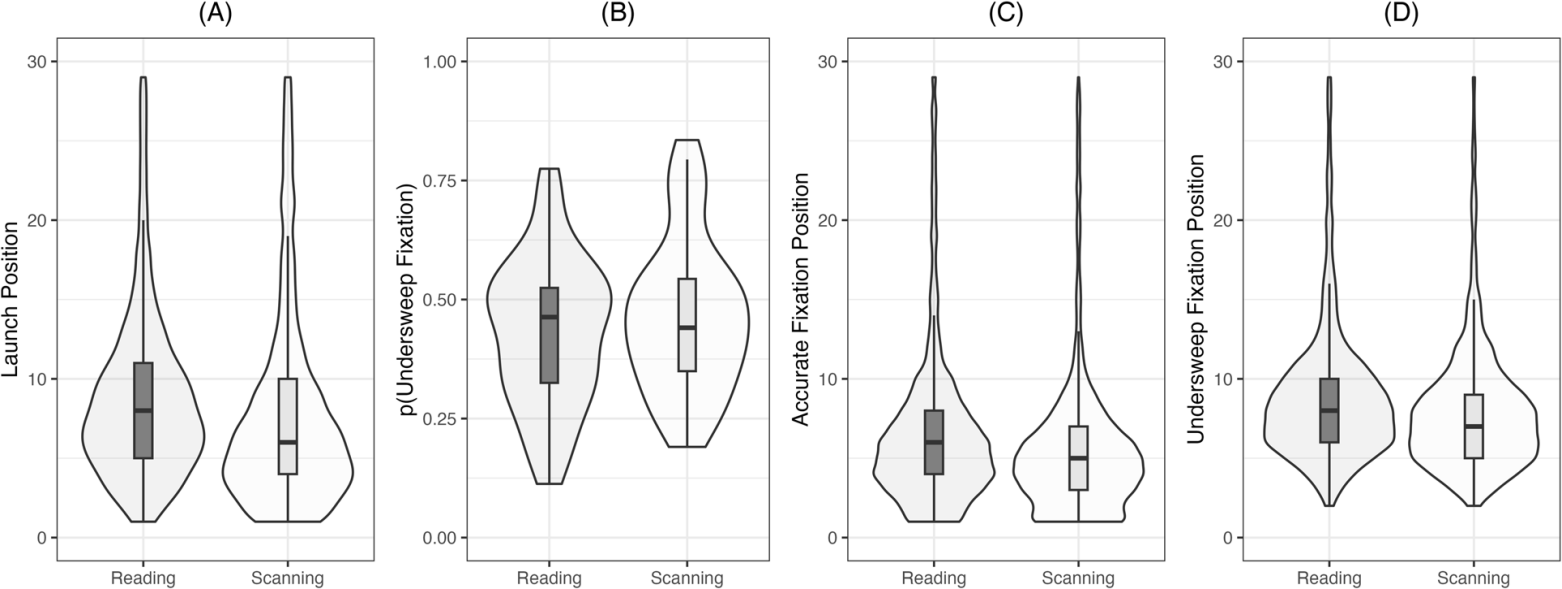
Return-sweep parameters: **A** Return-sweep launch position (characters from the end of a line) **B** Proportion of under-sweep fixations; **C** Accurate return-sweep landing position (characters from the start of the line); and **D** Under-sweep landing position (characters from the start of the line). The box extends from the first to the third quartile, with the line in the middle representing the median

We report exploratory, non-registered analyses for four return-sweep and corrective saccade parameters. Descriptive statistics are reported in Table 3, and distributions are visualized in Fig. 3.

First, for return-sweep launch position, a linear mixed-effects model (*lmer*(*dv*$\sim$ *Task + (1 + Task / Participant) + (1 / Item)*)) indicated no significant difference between tasks (see Table 4). The Bayes factor for the fixed effect of Task indicated that there was insufficient evidence to draw a decisive conclusion. Second, for the probability of making an under-sweep fixation, the generalized linear mixed-effects model (*glmer*(*dv*$\sim$ *Task + (1 + Task / Participant) + (1 + Task / Item)*)) indicated no significant difference between tasks, with Bayes factors favoring the null. Third, for accurate line-initial landing position, the linear mixed-effects model (*lmer*(*dv*$\sim$ *Task + (1 + Task / Participant) + (1 / Item)*)) indicated that readers’ accurate line-initial fixations landed closer to the left margin for scanning than text reading. Finally, under-sweep landing position, the linear mixed-effects model (*lmer*(*dv*$\sim$ *Task + (1 + Task / Participant) + (1 / Item)*)) indicated no significant difference between tasks. The Bayes factor indicated insufficient evidence to draw a decisive conclusion.

## Discussion

Research on return-sweeps has produced the widely replicated findings that line-final fixations are shorter than intra-line fixations, accurate line-initial fixations are longer than intra-line fixations, and under-sweep fixations are shorter than intra-line fixations (e.g., Abrams & Zuber, 1972; Adedeji et al., 2022). There has been much discussion around the mechanisms underlying these return-sweep fixation duration differences, with explanations generally clustering on lexical processing or oculomotor/visual accounts.

To examine linguistic and oculomotor/visual processing contributions to return-sweep fixation durations, 41 participants read 30 passages of text for comprehension and scanned 30 *z*-letter strings for *x*s. First, we replicated the well-established finding that relative to intra-line reading fixations, line-final fixations and under-sweep reading fixations are shorter in duration, while accurate line-initial reading fixations are longer (e.g., Abrams & Zuber, 1972; Adedeji et al., 2022). Second, we compared fixation duration differences between intra-line fixations and return-sweep fixations across reading and scanning. The novel contributions of our work can be summarized in three general points. First, the reduction in line-final fixation durations, relative to intra-line fixations, did not differ across tasks. Second, the increase in fixation durations for accurate line-initial fixations, relative to intra-line fixations, was smaller during scanning than reading. Third, the reduction in under-sweep fixation durations, relative to intra-line fixations, was larger during scanning than reading. Broadly, these results indicate the limited role of lexical processing in determining line-final fixation durations, that both oculomotor/visual and linguistic processing determine accurate line-initial fixation durations, and that oculomotor processing may determine under-sweep fixation durations. We further discuss each point in turn.

**Table 3:** Mean return-sweep and corrective saccade parameters per task

Parameter	Reading	Scanning
Launch position	8.79 (5.33)	7.80 (6.14)
p(Under-sweep fixation)	0.42 (0.49)	0.46 (0.50)
Accurate landing position	6.61 (4.93)	5.56 (4.46)
Under-sweep landing position	8.84 (4.30)	8.00 (4.00)

Note: ^a^Standard deviations are shown in parentheses

**Table 4:** Linear mixed-effects results and Bayes factors for return-sweep and corrective saccade parameters

Measure	Fixed effect	b	SE	t/z	BF10
Launch position	(Intercept)	8.40	0.36	23.17	−
	Task	−0.56	0.39	−1.43	0.87
p(Under-sweep fixation)	(Intercept)	−0.33	0.11	−2.96	−
	Task	0.18	0.12	1.48	0.33
Accurate landing position	(Intercept)	6.80	0.29	23.50	−
	Task	−1.27	0.33	−3.79	72.99
Under-sweep landing position	(Intercept)	8.63	0.31	27.60	−
	Task	−0.50	0.34	−1.45	1.03

Note: ^a^The Bayes factor for p(Under-sweep fixation) was rounded up from 0.325

Our mixed-effects analysis of data from both tasks replicates a widely reported finding in the literature that intra-line fixations are longer during scanning than reading (e.g., Al-Zanoon et al., 2017; Rayner & Fischer, 1996). However, what is also clear from the data is that this increase in fixation duration extends to return-sweep fixations, where line-final, accurate line-initial and under-sweep fixations were longer during scanning than reading. Effectively, this constitutes a main effect of task type. While the theoretical interpretation of longer fixation durations in z-reading continues to be debated (Al-Zanoon et al., 2017; Nuthmann et al., 2007; Rayner & Fisher, 1996; Reichle et al., 2010) one interpretation (Vitu et al., 1995) is that fixations are longer during scanning because word identification occurs more rapidly compared to the detection of a target letter.

When comparing the difference between intra-line fixations and line-final fixations across tasks, the reduction in duration did not differ between reading and scanning. Given that the same reduction occurred under two tasks with different lexical processing demands, it would suggest a limited role of lexical processing in determining these fixation durations and points towards shorter line-final fixations being driven by oculomotor or visual processing. Given that both tasks have similar oculomotor and visual processing demands, we cannot pinpoint the exact cause of shorter line-final fixations. Previously, Hofmeister (1998) suggested that the primary purpose of a line-final fixation is to program the return-sweep. This may indeed be the case but, given previous evidence (Parker et al., 2023; Parker & Slattery, 2024), it is clear that this does not mean that lexical processing is reduced or absent during a line-final fixation.

Our results indicated that the increase in duration for accurate line-initial fixations, relative to intra-line fixations, was smaller during scanning than during reading. A supplemental model fitted to the scanning data confirmed that, while this increase was smaller for scanning, accurate line-initial fixations were still longer than intra-line fixations. If longer accurate line-initial fixations during reading were driven purely by oculomotor/visual processing, we might have expected the same increase for accurate line-initial fixations across both tasks. By contrast, if this increase stemmed from the processing of meaningful linguistic content, then we may have expected a $Fixation~Type~\times ~Task$ interaction in our pre-registered model and no difference in fixation duration between accurate line-initial fixations and intra-line fixations in our supplemental model fitted to scanning data. The data supported neither of these predictions. The most parsimonious explanation here is then that both linguistic and oculomotor/visual processing contribute to longer accurate line-initial fixations. Perhaps this reflects a combination of saccade planning and delayed lexical access driven by the lack of parafoveal preview prior to direct fixation, where saccade planning adds a penalty onto both reading and scanning, and delayed parafoveal processing adds an additional penalty onto reading only.

Our comparative analysis of data from both tasks also indicated that the reduction in under-sweep fixation durations, relative to intra-line fixations, was larger during scanning than reading. Given the evidence that lexical processing for line-initial words can occur during an under-sweep and that readers can acquire useful information that informs subsequent reading of words receiving an under-sweep fixation (Parker & Slattery, 2019; Parker et al., 2020; Slattery & Parker, 2019), it may be that this slows down the rapid deployment of a corrective saccade during reading and this is why the difference in duration between intra-line and under-sweep fixations is larger during scanning as compared to reading. Of course, this is not the only interpretation of the data. There has been discussion that corrective saccades are driven by visual feedback following a saccade (Prablanc, Massé, & Echallier, 1978, Prablanc & Jeannerod, 1975), and it has also been reported that corrective saccade latencies in non-reading tasks are shorter when saccades land farther from their intended target (Becker, 1972). Given that participants’ accurate line-initial fixations during scanning were closer to the left margin, it may be that scanning requires a more meticulous encoding strategy where participants target the very start of a line whereas they are targeting the preferred or optimal viewing location during reading (McConkie et al., 1989; Rayner, 1979), which is further from the left margin. If this were the case, retinal feedback during scanning would more rapidly indicate a deviation from the intended location of the saccade even when under-sweep landing positions were comparable, resulting in shorter under-sweep fixations during scanning than reading. However, the fact that under-sweep fixation durations were still longer in scanning compared to reading is difficult to reconcile with this. Such an explanation is also made weaker by evidence showing that return-sweep landing sites are generally unrelated to the length of the first word on a line (Slattery & Vasilev, 2019), suggesting that readers target the very left margin under both tasks. Of course, it is important not to over-interpret this effect. While the null hypothesis evidence suggests a statistically reliable difference in the reduction in fixation duration for under-sweep fixations across tasks, the Bayesian evidence favors the null. Currently, there is little consensus on interpreting such a pattern of results, but they suggest that this difference may be small at best.

It is also important to mention some limitations of using the *z-reading paradigm* as a control condition to reading. The *z-reading paradigm* preserves the text’s spatial layout whilst removing higher-level linguistic information. However, one drawback is that visual complexity may differ between reading and scanning. As such, it could be argued that visual processing may be easier during scanning because of the less varied input. Therefore, any differences between reading and scanning may reflect differences in visual complexity. It is also important to note that task demands differ between reading and scanning beyond the additional need for higher-level linguistic processing during reading. Previous research has shown that readers adopt sophisticated strategies to accommodate task demands. For example, Kaakinen and Hyönä (2010) reported larger frequency effects when participants were proofreading than when reading for comprehension, and Schotter et al. (2014) reported that predictability effects increased when proofreading required successful integration of individual words with a sentence context. Thus, it is conceivable that the differences observed between fixation durations during reading and scanning in part reflect something about the reader’s goal in addition to a lack of higher-order linguistic processing. However, it should be noted that task demands had very little influence on return-sweep landing positions and the frequency of corrective saccades in a study where participants scanned strings of letters and numbers and were asked to either subvocally recite numbers, count how often the number 6 appeared in the stimuli, or add odd numbers together (Hofmeister, 1998). This evidence indicates that task demand effects on return-sweep behavior may be minimal. That said, to disentangle the effects of language processing and task demands, future research could additionally maintain lexical content while varying task demands and compare return-sweep fixations during reading, scanning, and proofreading.

To conclude, our research illustrates the remarkable consistency of the oculomotor system, given that across both reading and scanning line-final and under-sweep fixations were shorter than intra-line fixations, while accurate line-initial fixations were longer. While the basic pattern of return-sweep differences was observed for both tasks, there were nuanced differences. The reduction in line-final fixations did not differ across tasks, enabling us to conclude that shorter line-final fixation durations during reading cannot be attributed to higher-level lexical processing. Instead, it is likely driven by oculomotor or visual processing. By contrast, there was evidence that the increase in duration for accurate line-initial fixations was smaller during scanning, suggesting these fixations likely reflect a combination of both linguistic and oculomotor processes.

## Supplementary Information

Below is the link to the electronic supplementary material.

Supplementary file 1 (pdf 95 KB)

## Data Availability

The materials and the data sets generated and analyzed are available in the Open Science Framework (OSF) repository, https://osf.io/tpf8e/?view_only=96406d577b8241aeb98c1b0785349580. This repository also includes an R Markdown script to reproduce all analyses and generate the manuscript.

## Availability of data and materials

The materials and the data sets generated and analyzed are available in the Open Science Framework (OSF) repository, https://osf.io/tpf8e/?view_only=96406d577b8241aeb98c1b0785349580.

## Code Availability

The R Markdown script to reproduce all analyses and generate the manuscript are available in the Open Science Framework (OSF) repository, https://osf.io/tpf8e/?view_only=96406d577b8241aeb98c1b0785349580.
