# Supplementary material for: Are the differences between intra-line and return-sweep fixation durations driven by linguistic, oculomotor, or visual processing? A comparison of eye movements during reading and z-string scanning
Source: Psychon Bull Rev. 2025 Jul 25;32(6):3055–66. doi: 10.3758/s13423-025-02738-x (PMC12627124; doi:10.3758/s13423-025-02738-x)
Supplement: Supplementary file 1 — (pdf 95 KB) [file 13423_2025_2738_MOESM1_ESM.pdf]

Accurate line-initial fixations but not line-final fixations differ from intra-line fixations during both reading and z-string scanning

Online Supplemental Materials

Adam J. Parker, Muchan Tao, Martin R. Vasilev

Distribution of *x*s in Experimental Items

For z-string stimuli, the letter *x* was randomly inserted 5-15 times in the string of *z*s. Here, we report an additional analysis examining the spatial location of *x*s in these items. This was achieved by calculating the relative position of each *x* on a given line of text, to account for some lines being longer than others. We then divided the line into three equal portions: start (relative position 0.00 - 0.33), middle (relative position 0.34 - 0.66), and end (relative position 0.67 - 1.00).

Across all items, there were 77 *x*s in the first third (start) of the line, 92 *x*s in the second third (middle) of the line, and 84 *x*s in the last third (end) of the line. To statistically examine whether there was a reliable difference in the number of *x*s across the three regions, we conducted an item level analysis using a linear regression and report the pairwise comparisons using the *emmeans()* function from the emmeans package (version 1.10.6; Lenth et al., 2024). Pairwise comparisons indciated that the count of *x*s per item did not differ between the start and middle (b= -0.32, SE= 0.39, t= -0.81, p= 0.700), the start and end (b= -0.25, SE= 0.40, t= -0.63, p= 0.806), or the middle and end (b= 0.07, SE= 0.39, t= 0.17, p= 0.984). Based on this analysis, we have faith that the effects observed are not simply a result of *x*s appearing in extreme locations.
